# Quality Evaluation of Cone Biopsy Specimens Obtained by Large Loop Excision of the Transformation Zone

**DOI:** 10.14740/jocmr1951w

**Published:** 2015-02-09

**Authors:** Aristoteles Mauricio Garcia Ramos, Erika Souza Garcia Ramos, Helena Lucia Barroso dos Reis, Ricardo Bueno de Rezende

**Affiliations:** a Santa Casa de Misericordia School of Science (EMESCAM), Vitoria, ES, Brazil; b Espirito Santo Federal University (UFES), Vitoria, ES, Brazil

**Keywords:** Conization, Cervical intraepithelial neoplasia, Colposcopy, HPV

## Abstract

**Background:**

Large loop excision of the transformation zone (LLETZ) has been used for the diagnosis and treatment of precancerous cervical lesions, and it is the first choice of treatment in the majority of cervical pathology services. The aim of this study was to evaluate the presence of thermal artifacts, the need for serial sections, the percentage of clear and involved resection margins and the relationship between endocervical gland involvement and the severity of the lesion in samples resected using LLETZ.

**Methods:**

A retrospective study was performed at Santa Casa de Misericordia School of Science (HSCMV), Vitoria, Espirito Santo, Brazil with a sample of 52 histopathology slides from patients submitted to conization because of abnormal cytology findings and a biopsy result of cervical intraepithelial neoplasia (CIN) 2, CIN 3 and adenocarcinoma *in situ*. Statistical analysis was performed using Student’s *t*-test.

**Results:**

Serial sections were required to confirm diagnosis in four of 52 cases. Thermal artifacts were present in all cases, with grade I being the most common (94.2% of cases). Clear margins were found in 96.2% of cases. No association was found between glandular involvement and CIN 1 (P > 0.05); however, there was an association with CIN 2 and CIN 3 (P < 0.05).

**Conclusion:**

The amount of excised tissue was sufficient, thermal artifacts were slight, resection margins were clear in most of cases, and a possible association was found between glandular involvement and the severity of the lesion.

## Introduction

The National Cancer Institute (INCA) estimates indicate more than 15,000 new cases of cervical cancer occurring in 2014 [1].

The decline in the incidence of invasive cervical cancer is attributed to an increase in early detection and treatment of precursor lesions. This has been attained through the implementation of routine cervical cytology. Patients with abnormal cytology are referred to a specialist center to be submitted to colposcopy and, possibly, cervical biopsy.

According to the Bethesda classification, potentially premalignant squamous lesions are divided into three categories: atypical squamous cells (ASCs), low-grade squamous intraepithelial lesions (LSILs) and high-grade squamous intraepithelial lesions (HSILs). The LSIL classification includes cervical intraepithelial neoplasia (CIN) type 1 (mild dysplasia) and human papilloma virus (HPV)-related abnormalities. HSIL includes CIN 2 and CIN 3 (moderate and severe dysplasia, respectively) and carcinoma *in situ* and the grade of the lesion may present a linear correlation between the various HPV types [2]. The majority of low-grade lesions caused by high-risk HPV are transitory and normally regress spontaneously [3].

Whenever cervical cytology suggests the presence of HSIL, colposcopy and directed biopsy should be performed [4]. Upon histopathology confirmation, the treatment of choice for CIN 2 or 3 is surgical technique called the loop electrosurgical excision procedure (LEEP) [5, 6], also known as large loop excision of the transformation zone (LLETZ) or radiosurgery [6], referred to in Brazil as high-frequency surgery [7].

Prendiville began to use LLETZ and reported the multiple advantages of this new technique of performing cervical lesion biopsies compared to conventional biopsies [8].

LLETZ, both for the diagnosis and treatment of precancerous cervical lesions, is the first choice of treatment in the majority of cervical pathology services [9].

Specimens obtained by cone biopsy allow histopathology to be performed with a high degree of accuracy, permitting an assessment of whether the resection margins are affected and whether there is glandular involvement, which are risk factors for the recurrence of CIN [10].

Cone biopsy allows simultaneous diagnosis and treatment of the lesion, which is impossible with destructive local methods [11-13].

LLETZ is considered the first choice for treatment in view of its efficacy, low cost and low rate of complications [5, 14, 15]. The classic indications for cold-knife cone biopsy are: positive endocervical curettage, two degrees of discrepancy between cytology and biopsy findings, and an unsatisfactory colposcopy [16].

Evaluating the use of LLETZ in precursory cervical lesions, this study analyzed the presence of thermal artifacts, the need for serial sections, the percentage of clear and involved resection margins and the association between endocervical glandular involvement and the severity of the lesion in the excised samples.

## Materials and Methods

The protocol of the present study was approved by the internal review board of the Santa Casa de Misericordia School of Science in Vitoria (EMESCAM) under reference number 088/2011. Informed consent was not obtained due to the retrospective nature of the investigation conducted in archival biopsy specimens.

Fifty-two histopathology slides from patients who had been submitted to LLETZ between August 2003 and October 2007 at the teaching hospital of the Santa Casa de Misericordia School of Science were evaluated retrospectively.

In the cases selected, the women had received colposcopy results considered abnormal according to the 2001 Bethesda classification, with biopsy findings of CIN 2, CIN 3, and adenocarcinoma *in situ*. They were referred for cervical cone biopsy. Directed biopsies were performed using Gaylor Medina forceps. Cone biopsies were carried out by LLETZ, guided in accordance with the colposcopy findings and Schiller’s test results, and performed by the same surgeon in all cases and using the same type of equipment so as to minimize the incidence and extent of thermal damage and involvement of the resection margins in the procedures.

The tissue samples (cones) were fixed in formalin and marked at the 12 o’clock position, and then sent to the institution pathology laboratory, where they were all analyzed by the same pathologist. At macroscopy, the entire anterior labium and the endocervical margin were identified with black ink, thus obtaining the longitudinal, cross-sectional and anteroposterior measurements. The mean thickness of the sections was 4 mm. Sections were embedded in paraffin blocks and stained with hematoxylin and eosin. Data on the severity of the lesion and its size, whether there was involvement of glandular openings or crypts, the presence of thermal artifacts, and whether or not the resection margins were involved were also collected.

The qualitative thermal alterations were divided into three grades: slight (I), moderate (II) and severe (III) according to the criteria defined by Messing et al in 1994 [17]. Grade I is defined as a slight thermal alteration that allows histological evaluation to be performed without any difficulty, the surgical margins to be identified with precision and the severity of the neoplasia to be established. Grade II is defined as the presence of moderate thermal alterations that result in relative difficulty both in correctly interpreting the severity of the neoplasia and in establishing the actual involvement of the resection margins. Finally, the samples with intense thermal damage or loss of epithelium in which histopathological evaluation either of the severity of the neoplasia or of the surgical margins proved impossible were classified as grade III [17]. The need for serial sections to enable a final diagnosis to be reached was also evaluated.

Data were analyzed using Student’s *t*-test and the significance level was established at P < 0.05. The SPSS Statistical Software Program, version 11.5 for Windows, was used for the statistical analysis.

## Results

Of the 52 slides evaluated, only four required serial sections to reach diagnosis (Table 1), showing that the routinely used conventional histology section is sufficient for the purpose.

**Table 1:** Need for Serial Sections

	Number of cases	Percent
No	48	92.3
Yes	4	7.7
Total	52	100

As shown in Table 2, analysis of the qualitative results of the thermal artifacts showed that these were present in every case, with grade I being the most common, corresponding to 94.2% of cases. Grade II was present in 5.8% of the samples and there were no cases of grade III.

**Table 2:** Presence and Degree of Thermal Artifacts

	Number of cases	Percent
Slight	49	94.2
Moderate	3	5.8
Severe	0	0
Total	52	100

Table 3 shows that in the present study, the surgical margins were clear in 96.2% of cases, with the margins being involved in only two samples, corresponding to 3.8%.

**Table 3:** Surgical Margin Status

	Number of cases	Percent
Clear	50	96.2
Involved	2	3.8
Total	52	100

As shown in Table 4, there may be an association between the involvement of glandular openings and the degree of severity of the lesion. The results of the test showed no association between glandular involvement and CIN 1 (P > 0.05), but a positive association with CIN 2 and with CIN 3 (P < 0.05).

**Table 4:** Involvement of Glandular Openings Versus CIN 1, CIN 2, and CIN 3

	Involvement of glandular openings		Total	P-value
	Yes	No		
CIN 1				
Yes	0 (0%)	2 (3.8%)	2 (3.8%)	
No	25 (48.1%)	25 (48.1%)	50 (96.2%)	0.491
Total	25 (48.1%)	27 (51.9%)	52 (100%)	
CIN 2				
Yes	0 (0%)	12 (23.1%)	12 (23.1%)	
No	25 (48.1%)	15 (28.8%)	40 (52%)	0.000
Total	25 (48.1%)	16 (44.4%)	52 (100%)	
CIN 3				
Yes	25 (48.1%)	12 (23.1%)	37 (71.2%)	
No	0 (0%)	15 (28.8%)	15 (28.8%)	0.000
Total	25 (48.1%)	27 (51.9%)	52 (100%)	

CIN: cervical intraepithelial neoplasia.

## Discussion

### Main findings

The qualitative findings with respect to thermal artifacts showed that they were present in all cases, with grade I being the most common, corresponding to 94.2% of all cases. In 5.8% of samples, thermal artifacts were considered grade II; however, none of the cases were considered grade III, showing that thermal artifacts do not interfere with the histological evaluation of lesions.

### Strength and limitations

In the present study, the resection margins were clear in 96.2% of the cases, with margins being affected in only two samples (3.8%). The risk factor for recurrence of CIN that has been most evaluated is whether or not the resection margins of the sample are affected [10]. We strictly limited our population to a public university hospital. However, we recognize that these outcomes may not be representative of other settings.

### Interpretation

These results obtained with respect to grade III are in agreement with, or even better than, those reported by many authors [17, 18]. Conflicting findings have been published in the literature with respect to the thermal damage caused by LLETZ. Montz et al reported that this procedure results in a high rate of tissue damage to the resection margins, leading to relative limitations in reaching an accurate histopathological interpretation [18]. Nevertheless, other studies affirm that resection margins are found to be satisfactory in the samples obtained with LLETZ, permitting adequate evaluation [19]. This was confirmed in the study conducted by Taha et al, in which thermal damage was found in all the samples obtained from patients submitted to LLETZ; however, in 91% of cases this damage was insignificant and histopathology was able to be conducted adequately [9]. In the study carried out by Messing et al, thermal damage was attributed to a high cutting power, the type of wave, moving the loop too slowly, stopping the procedure during excision, the pressure of the electrode on the tissue and the use of inappropriate or carbonized electrodes [17].

In the present study, the resection margins were clear in 96.2% of the cases, with margins being affected in only two samples (3.8%). The risk factor for recurrence of CIN that has been most evaluated is whether or not the resection margins of the sample are affected [10]. In fact, it is known that clear margins are not necessarily indicative of cure and, furthermore, that if the margins are affected this is not necessarily indicative of residual disease [20, 21]. Resected margins may be positive in up to 48% of cases, [22] and ectocervical margins, endocervical margins or both may be involved. When the endocervical margins are affected, this is of greater concern, since they may be indicative of an invisible form of the disease. Furthermore, in many cases, lesions of the ectocervical margin may be destroyed by cauterization at the borders [23]. Complete histological evaluation of the resected tissue with LLETZ cannot be considered a major predictor of persistence or recurrence of the disease [24]; hence, the risk of residual or recurrent disease following cone biopsy remains unclear [25]. Some authors suggest that patients who were positive for HPV 16 infection ought to be followed up closely after treatment [26]. However, it is important to emphasize that after resection of the tissue, cauterization of the entire resulting crater should be performed, which may destroy any residual lesion [10].

Greater value should be given to the presence of glandular involvement in the surgical sample rather than to a finding of margin involvement when considering the likelihood of recurrence of the lesion. Therefore, the importance of evaluating whether there is glandular involvement in the cone biopsy samples is clear [27]. In the present study, a possible association was found between the involvement of glandular openings and the severity of the lesion. Analysis showed that there is no association between glandular involvement and CIN 1 (P > 0.05); however, an association was found between glandular involvement and CIN 2 and CIN 3 (P < 0.05). Dysplastic cells may remain in the endocervical glands covered by the normal epithelium and may progress to more advanced grades of dysplasia or even invade the cervical stroma, even when no abnormalities are detected at cytology or colposcopy. This phenomenon may explain the finding of invasive carcinoma in patients previously submitted to cone biopsy and who have a sequence of normal cytology tests [27].

In this study the principal risk factors for a recurrence of CIN were analyzed, with results showing the amount of the excised tissue to be adequate, since few cases required additional histological sections. Thermal artifacts were present in the majority of the samples; however, most were considered grade I. The resection margins were clear in most cases; however, this does not guarantee the absence of residual lesion.

To assure a surgical specimen of good quality that will permit adequate histopathological evaluation, the surgeon must be skilled in the LLETZ technique and the appropriate loops must be selected.

### Key message

In this study the histopathology analysis of patients undergoing LLETZ due to abnormal cytology presented sufficient excised tissue with few thermal artifacts, clear resection margins in most cases and a possible association between glandular involvement and the lesion severity.

## Abbreviations

LLETZ: large loop excision of the transformation zone
CIN: cervical intraepithelial neoplasia
HPV: human papilloma virus
